# Electrochemical Aptasensor for Myoglobin-Specific Recognition Based on Porphyrin Functionalized Graphene-Conjugated Gold Nanocomposites

**DOI:** 10.3390/s16111803

**Published:** 2016-10-28

**Authors:** Guojuan Zhang, Zhiguang Liu, Li Wang, Yujing Guo

**Affiliations:** 1 Institute of Environmental Science, Shanxi University, Taiyuan 030006, China; 201422901011@email.sxu.edu.cn (G.Z.); liuzg@sxu.edu.cn (Z.L.); wangli@sxu.edu.cn (L.W.); 2 College of Chemistry and Chemical Engineering, Shanxi University, Taiyuan 030006, China

**Keywords:** graphene, TCPP, AuNPs, myoglobin

## Abstract

In this work, a novel electrochemical aptasensor was developed for sensitive and selective detection of myoglobin based on meso-tetra (4-carboxyphenyl) porphyrin-functionalized graphene-conjugated gold nanoparticles (TCPP–Gr/AuNPs). Due to its good electric conductivity, large specific surface area, and excellent mechanical properties, TCPP–Gr/AuNPs can act as an enhanced material for the electrochemical detection of myoglobin. Meanwhile, it provides an effective matrix for immobilizing myoglobin-binding aptamer (MbBA). The electrochemical aptasensor has a sensitive response to myoglobin in a linear range from 2.0 × 10^−11^ M to 7.7 × 10^−7^ M with a detection limit of 6.7 × 10^−12^ M (S/N = 3). Furthermore, the method has the merits of high sensitivity, low price, and high specificity. Our work will supply new horizons for the diagnostic applications of graphene-based materials in biomedicine and biosensors.

## 1. Introduction

Myocardial infarction has become a leading cause of death in most industrialized nations. The prevention or treatment of acute myocardial infarction (AMI) for people has become especially urgent, and accurate diagnosis of the patients is essential coequally [1]. It is worth mentioning that myoglobin is the main marker of early acute myocardial infarction as the concentration of myoglobin could indicate myocardial damage [2]. Generally, the normal concentration of myoglobin in the human body ranges from 0.48 nM to 0.90 nM, which is much lower compared to that in cardiac vascular disease patients. Myoglobin is quickly released into circulation within several hours after AMI onset, and myoglobin concentration will ultimately be elevated to 4.8 μM [3]. Therefore, sensitive and convenient detection of myoglobin is of great value. To date, methods that have been reported for measuring myoglobin include mass spectrometry [4], surface plasmon resonance [5], and electrochemical [6] and colorimetric biosensors [7]. Although most of the methods possess high sensitivity and selectivity, there still exist some limitations, such as expensive equipment and complicated operation [8]. In order to surmount these shortcomings, scientists are making efforts to develop simple, rapid, and efficient analytic techniques.

Electrochemical sensors have the advantages of simplicity, low cost, and the possibility to be constructed as portable devices for on-site determination [9,10]. Therefore, an increasing number of electrochemical sensors have been developed for myoglobin detection, including the three-dimensional reduced graphene oxide and gold composite with carbon ionic liquid electrode (3D RGO–Au/CILE) composite sensor [6], molecularly imprinted polymer/gold on screen printed electrode (MIP/Au–SPE) devices [11], and peptide-based electrochemical myoglobin [12]. Among them, electrochemical aptasensors and immunosensors are much superior due to their high specificity and sensitivity resulting from high affinity between aptamers/antibodies and the corresponding targets [13,14]. However, antibodies are usually costly, difficult to synthesize, and unstable in the long term. Thus, their applications in the construction of biosensors are limited. Compared with antibodies, aptamers can be synthesized chemically with ease and extreme accuracy at low cost. Furthermore, they are easy to modify and show excellent stability for long-term storage. Therefore, they have been widely used to construct biosensors recent years [15].

The other important factor to improve the sensitivity of the biosensors is the material of the modified electrode. Graphene, a two-dimensional carbon crystal with only one atom thickness, has drawn much attention because of its excellent electrical conductivity [12,16,17], high surface-to-volume ratio, and remarkable chemical stability [18]. The unique characteristics of graphene make it an excellent material for electrochemical sensors [19,20]. In particular, the combination of graphene with nanoparticles has been exploited in the development of various biosensing platforms because they have displayed synergic effects with graphene and other nanoparticles [21]. Among the diverse nanoparticles, gold nanoparticles (AuNPs) have obtained the most extensive and in-depth study due to their unique properties including excellent biocompatibility [22], high electrical conductivity and catalytic activity [23,24,25], easy preparation, and good stability [26]. Furthermore, upon introducing AuNPs into the graphene system, the aptamer will be able to bind on the electrode surface by forming a Au–S bond between thiolated aptamer and AuNPs [27,28]. So, the incorporation of AuNPs with graphene will not only enhance the electron transfer and amplify the electrochemical signals, but also provide binding sites for thiolated aptamer immobilization [29].

In this study, meso-tetra (4-carboxyphenyl) porphyrin functionalized graphene conjugated gold nanoparticles (TCPP–Gr/AuNPs) were synthesized via a simple wet-chemical strategy and self-assembly approach. TCPP is one of the anion porphyrins which can bind graphene through π–π stacking and improve the solubility and stability of graphene [30]. Moreover, it can be used as catalyst to enhance electrochemical reactions [31]. Based on the obtained nanocomposite, a sensitive electrochemical aptasensor (MbBA/TCPP–Gr/AuNPs) was constructed for myoglobin detection through myoglobin-specific binding with aptamers on the surface of the modified electrode. The aptamer with ferrocene can be regarded as an electrochemical probe for target analysis. It attached to the electrode without a target. In the presence of myoglobin, myoglobin-binding aptamer (MbBA) on the electrode surface could recognize the target by the conformational change [32,33,34]. Therefore, ferrocene increased its distance from the electrode surface. The electron transfer rate was decreased, resulting in reduced ferrocene signal. From the changes of the peak current in the absence and presence of myoglobin, a convenient electrochemical aptasensor for myoglobin was developed with high sensitivity and specificity and a broad linear range. This method has great potential for the determination of myoglobin in clinical diagnostics.

## 2. Materials and Methods

### 2.1. Materials and Instruments

Graphite was purchased from Alfa Aesar, and TCPP from Tokyo Chemical Industry Co., Ltd. (Tokyo, Japan). Hydrazine hydrate solution (80 wt %) and ammonia solution (30 wt %) were obtained from the Third Chemical Reagent Factory (Tianjin, China). MCH (6-mercapto-l-hexanol), tris (2-carboxyethyl) phosphine hydrochloride (TCEP) and poly (dimethyl diallyl ammonium chloride) (PDDA, Mw = 400,000–500,000, 20 wt % in water) were purchased from Sigma-Aldrich Co. (St. Louis, MO, USA) HAuCl_4_ was obtained from Beijing Chemical Reagent Factory (Beijing, China). Myoglobin protein (from human heart tissue) was purchased from Abcam (USA). Bovine serum albumin (BSA), human serum albumin (HSA), hemoglobin (Hb), and carcinoembryonic antigen (CEA) were obtained from Sigma Co. (St. Louis, MO, USA). Blood samples were provided by a local hospital. The human myoglobin-binding aptamer (MbBA): 5ʹ-SH–CH2)6–CCC TCC TTT CCT TCG ACG TAG ATC TGC TGC GTT GTT CCG A–Fc-3ʹ [8,35] was purchased from Shanghai Sangon Biotech Co., Ltd.,(Shanghai, China) and dissolved in T-buffer (20 mM Tri-HCl, 0.10 M NaCl, 5.0 mM MgCl_2_, 10 mM TECP, pH = 7.4). All reagents were analytical pure, and water used throughout all experiments was purified with the Millipore system.

UV–vis absorption spectra were recorded on a U-3010 spectrometer (Hitachi, Ltd., Tokyo, Japan). Fourier-transformation infrared (FT-IR) spectra were recorded on a Shimadzu 8400S spectrometer (Shimadzu, Kyoto, Japan). The morphology of the nanocomposite was recorded on a JEOL-2100 TEM (Electron Ltd, Tokyo, Japan) with an accelerating voltage of 200 kV. X-ray photoelectron spectroscopy (XPS) measurement was performed on an ESCALAB-MKII 250 photoelectron spectrometer (VG Co., New York, NY, USA) with Al Kα X-ray radiation as the X-ray source for excitation. Electrochemical measurements were conducted on a CHI660C Electrochemical Workstation (Chen Hua Instruments Co., Shanghai, China) with a conventional three-electrode system composed of a bare or modified glassy carbon electrode (GCE, 3.0 mm in diameter) as the working electrode, and a platinum wire and a saturated calomel electrode (SCE) as the counter electrode and the reference electrode, respectively.

### 2.2. Synthesis of the TCPP–Gr and TCPP–Gr/AuNPs

The graphite oxide (GO) was synthesized from natural graphite powder by a modified Hummer′s method [36]. TCPP–Gr were prepared as follows: 2.5 mL of 1.0 mg/mL GO was mixed with 2.5 mL 1.0 mg/mL TCPP, then 100 μL of ammonia solution and 10 μL of hydrazine solution were added. The mixture was vigorously shaken for a few minutes and heated at 60 °C for 3.5 h. The stable black dispersion was obtained. The dispersion was filtered with a nylon membrane (0.22 μm) to obtain TCPP–Gr that can be dispersed readily in water by ultrasonication.

The TCPP–Gr/AuNPs were synthesized as follows: 125 μL of PDDA was added into 4.0 mL of 0.12 mg/mL TCPP–Gr aqueous dispersion in a 15 mL reaction system, stirred with a magnetic stir bar for 15 min, then 200 μL of 10 mg/mL HAuCl_4_ was added into the vial. After several minutes, 2.0 mL of 20 mM fresh NaBH_4_ solution was added to the mixing solution at room temperature, followed by another 30 min stirring. The obtained TCPP–Gr/AuNPs nanocomposites were accumulated by centrifugation and washed two times with deionized water.

### 2.3. Fabrication of the Sensing Interface

GCE was polished to a mirror-like surface with 0.05 μm alumina powder before modification, and then rinsed with ethanol and re-distilled water. The cleaned GCE was dried with high-purity nitrogen gas. Then, 8.0 μL of 0.25 mg/mL TCPP–Gr/AuNPs dispersion was carefully dropped on the surface of the pretreated GCE and dried under an infrared lamp to obtain the modified TCPP–Gr/AuNPs/GCE. Subsequently, the TCPP–Gr/AuNPs/GCE was dipped in 30 μL of 2.5 μM MbBA solution including 10 mM TCEP for 4 h at 4 °C in a refrigerator [15]. Then, the complex was immersed in 200 μL of 1.0 mM MCH for 0.50 h to block possible remaining active sites and avoid nonspecific adsorption [23]. Thus, the aptasensor of MbBA/TCPP–Gr/AuNPs/GCE was obtained. Then, the electrode was rinsed with ultrapure water and PBS for the measurement of myoglobin.

### 2.4. Electrochemical Detection of Myoglobin

The well-prepared electrode was incubated in different concentration of myoglobin solutions for 45 min at room temperature. Differential pulse voltammetry (DPV) was employed to express the response for the myoglobin by measuring the peak current changes. DPV measurements were performed by scanning the potential from 0 V to 0.70 V with the amplitude of 50 mV, pulse width of 0.050 s. All the electrochemical measurements were performed in 0.10 M PBS (pH 7.4).

## 3. Results and Discussion

### 3.1. The Principle of the Aptasensor

**Scheme 1:**
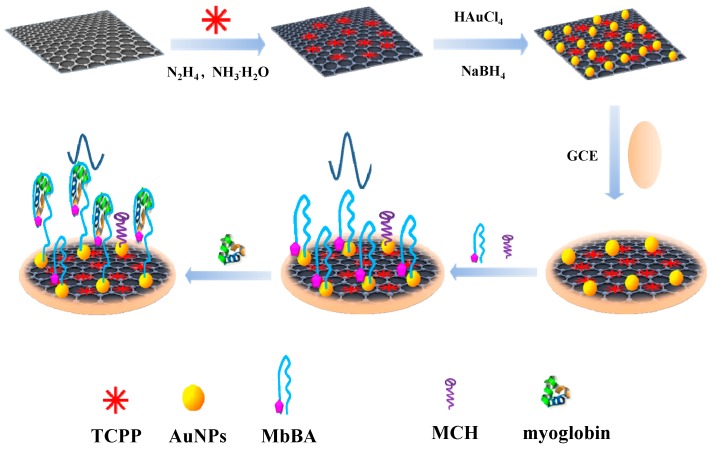
The procedure for the synthesis of meso-tetra (4-carboxyphenyl) porphyrin-functionalized graphene-conjugated gold nanoparticles (TCPP–Gr/AuNPs) nanocomposites and electrochemical aptasensor for myoglobin detection.

The procedure for the electrochemical sensing platform was shown in Scheme 1. Firstly, TCPP–Gr was prepared by a simple wet-chemical reaction under alkaline conditions. TCPP, a negatively charged water-soluble molecule with a large planar aromatic surface, could serve as a stabilizer for graphene through π–π stacking interactions. Then, AuNPs were incorporated into TCPP–Gr by fresh NaBH_4_ reduction and the MbBA was modified on the TCPP–Gr/AuNPs/GCE surface by Au–S bond [37]. The end of the MbBA with the thiol was combined with AuNPs, and the other end of the MbBA with ferrocene was attached to the electrode surface. Ferrocene served as an indicator for the detection of myoglobin. In the absence of myoglobin, ferrocene exhibited stronger signal. While in the presence of myoglobin, the MbBA on the electrode surface recognized the target specifically and the conformation of MbBA changed; simultaneously, ferrocene was far away from the electrode. As a result, the electron transfer rate was hindered, leading to the decrease of ferrocene signal [32,33,34]. The more myoglobin captured onto the electrode, the lower the peak current obtained will be. The decreased peak current is associated with the concentration of myoglobin. Consequently, an electrochemical aptasensor for myoglobin detection has been constructed.

### 3.2. Characterization of the TCPP–Gr/AuNPs

**Figure 1:**
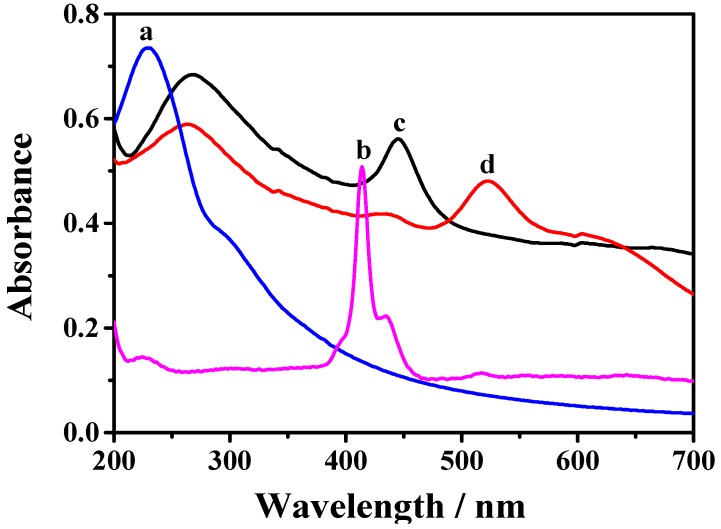
UV–vis spectra of graphite oxide (GO) suspension (a); TCPP (b); TCPP–Gr suspension (c); and TCPP–Gr/AuNPs suspension (d).

TCPP–Gr/AuNPs were firstly characterized by UV–vis spectroscopy. As shown in Figure 1, GO dispersion (curve a) exhibits a maximum absorption at 230 nm and a shoulder at 290–300 nm, corresponding to the π–π* transition of aromatic C–C bonds and the n–π* transition of the C=O bonds [38], respectively. TCPP (curve b) displays a narrow absorption at 414 nm which is the typical characteristic peak owing to the Soret band of TCPP and a series of weak peaks from 500 nm to 700 nm which are attributed to the Q-bands of TCPP. After GO was reduced by hydrazine to form TCPP–Gr, the absorption peak of GO shifted from 230 to 269 nm and a peak at 448 nm is observed, which corresponds to the Soret band of TCPP with a large bathochromic shift (34 nm). This illustrated that there were strong π–π interactions between graphene and TCPP [30]. After decorating AuNPs on the surface of TCPP–Gr, a new strong peak appeared at 530 nm, which was consistent with the surface plasmon of absorption of AuNPs. This demonstrated that TCPP–Gr/AuNPs were successfully synthesized (curve d).

**Figure 2:**
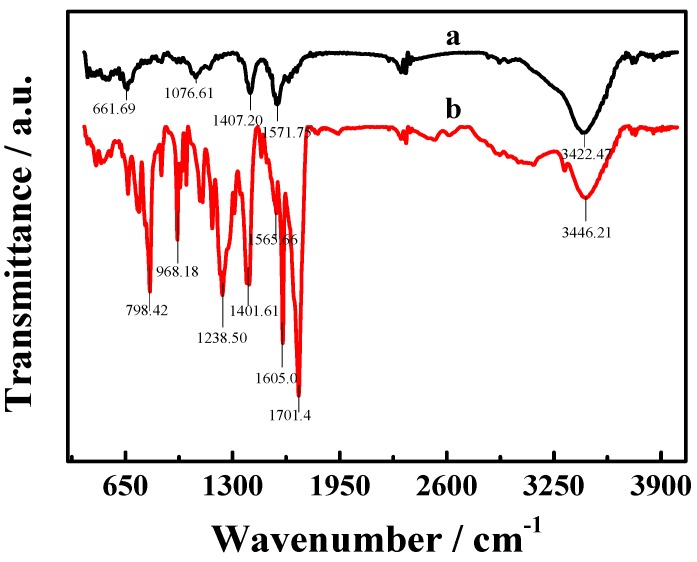
The Fourier-transformation infrared (FT-IR) spectra of TCPP–Gr (a) and TCPP (b).

FT-IR spectra (Figure 2) of TCPP–Gr (a) and TCPP (b) also provide an obvious verification for the successful synthesis of TCPP–Gr hybrids. The peak of 1701 cm^−1^ in TCPP is attributed to ν (C=O) vibrations of –COOH, whereas in TCPP–Gr it moved to 1572 cm^−1^ which due to the π–π stacking interactions between TCPP and graphene [30]. The spectrum of TCPP–Gr also demonstrated a broad, intense band at 3422 cm^−1^ which corresponded to O–H stretching vibrations of the O–H group from carboxyl as well as remaining water. In brief, FT-IR spectra illustrated that TCPP molecules are attached to the surface of on graphene.

**Figure 3:**
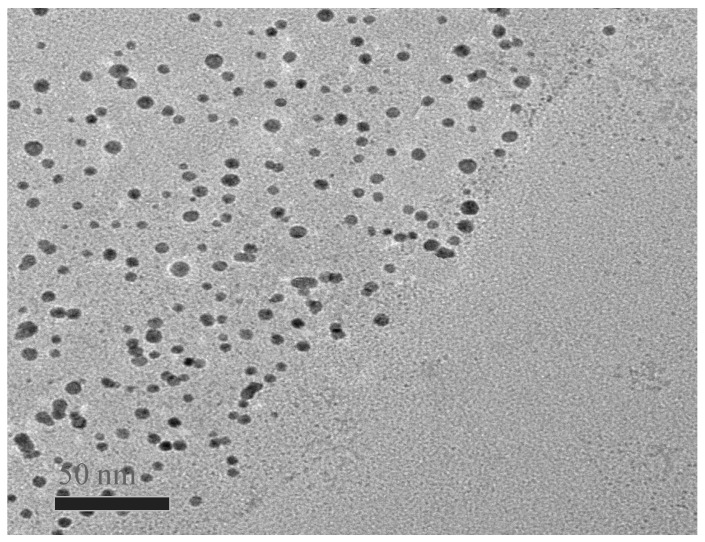
The TEM image of TCPP–Gr/AuNPs.

TEM was carried out to characterize the morphology of TCPP–Gr/AuNPs nanocomposites. As displayed in Figure 3, AuNPs evenly scattered on the surface of TCPP–Gr. The mean size of the AuNPs was estimated to be 18 nm.

**Figure 4:**
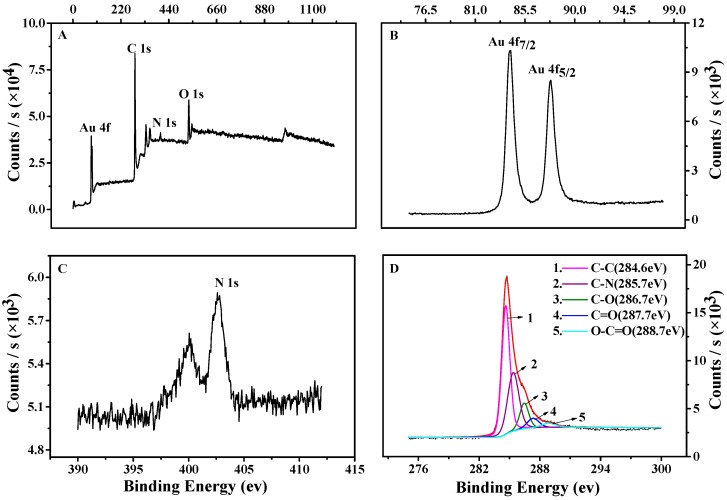
The X-ray photoelectron spectroscopy (XPS) spectra of TCPP–Gr/AuNPs (**A**); Au 4f (**B**); N 1s (**C**); and C 1s (**D**).

The constituent and oxidation states of TCPP–Gr/AuNPs nanocomposites were further analyzed by XPS, and results are shown in Figure 4. Compared to the graphene nanosheets, the survey of TCPP–Gr/AuNPs (Figure 4A) showed the presence of Au and N originating from AuNPs and TCPP, respectively. Figure 4B shows Au 4f_7/2_ and Au 4f_5/2_ peaks appeared at 84.4 eV and 87.8 eV, respectively. Figure 4C showed N 1s spectrum appeared at 402.5 eV, which originated from TCPP [39]. The C 1s spectrum of TCPP–Gr/AuNPs has five kinds of carbon bonds: C–C (284.6 eV), C–O (286.7 eV), C=O (287.7 eV), C=N (285.7 eV), and O–C=O (288.7 eV) (Figure 4D) [40,41]. All the above illustrated the formation of TCPP–Gr/AuNPs.

### 3.3. Characterization of the Aptasensor

**Figure 5:**
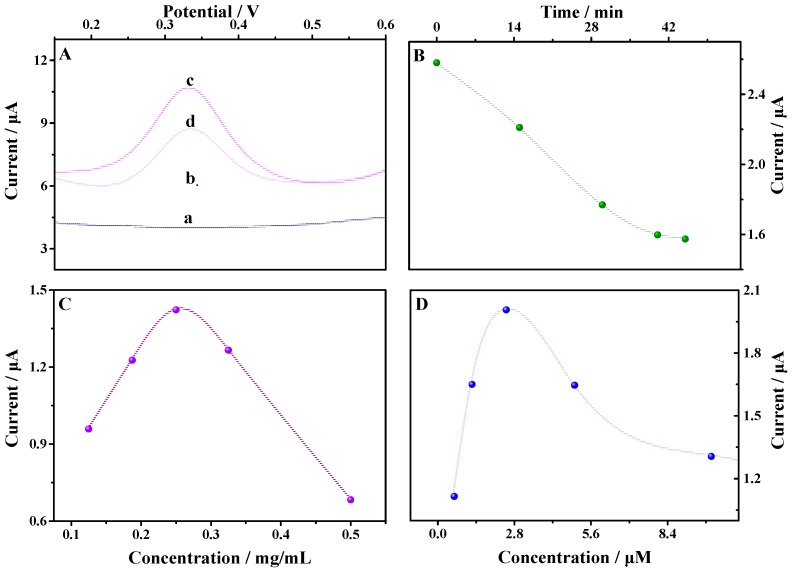
(**A**) Differential pulse voltammetry (DPV) of the bare electrode (curve a), TCPP–Gr/AuNPs/GCE (curve b), MbBA–Fc/TCPP–Gr/AuNPs/GCE (curve c), and MbBA–Fc/TCPP–Gr/AuNPs/GCE in 4.4 × 10^−7^ M myoglobin solution (curve d); (**B**) The relationship between the peak current and the incubated time; (**C**) The effect of the concentration of TCPP–Gr/AuNPs on the peak current; (**D**) The effect of the concentration of MbBA on the peak current (Amplitude: 50 mV, pulse width: 0.050 s).

DPV was employed to characterize the aptasensor. As can be seen from Figure 5A, there was no redox peak at the bare GCE (line a) and TCPP–Gr/AuNPs/GCE (line b) in the potential range. After modified MbBA was on the surface of the TCPP–Gr/AuNPs/GCE, a well-defined oxidation peak appeared at 0.33 V, which attribute to the ferrocene modified on the end of MbBA. The result indicated that the sensing interface of MbBA/TCPP–Gr/AuNPs/GCE (line c) was successfully constructed. After 4.40 × 10^−7^ M of myoglobin was added into the above buffer solution and incubated for 45 min, the peak current obviously decreased (line d) due to the impediment of electron transfer by myoglobin.

### 3.4. Optimization of Experimental Conditions

#### 3.4.1. Influence of the Incubation Time

Myoglobin should be captured on the surface of MbBA/TCPP–Gr/AuNPs/GCE by specific binding with the aptamer, and the incubation time is an important factor that affects the sensitivity. When MbBA/TCPP–Gr/AuNPs/GCE was immerged into 1.47 × 10^−10^ M myoglobin solution, DPV was used to monitor the changes of the signal. As shown in Figure 5B, the peak currents decreased evidently within 45 min and reached a plateau, which showed the saturation of bioaffinity between MbBA and myoglobin. As a result, 45 min was selected as the optimal incubation time for myoglobin detection.

#### 3.4.2. Influence of the TCPP–Gr/AuNPs Concentration

To optimize the amount of TCPP–Gr/AuNPs, 8.0 μL of TCPP–Gr/AuNPs solution with different concentrations was dropped onto the surface of GCE, respectively. Then, 30 μL of 2.5 μM MbBA solution was spread on the TCPP–Gr/AuNPs/GCE. The peak current increased with the increasing concentration of TCPP–Gr/AuNPs from 0.125 mg/mL to 0.25 mg/mL and then decreased when the concentration of TCPP–Gr/AuNPs exceeded 0.25 mg/mL (Figure 5C). Thus 8.0 μL of 0.25 mg/mL TCPP–Gr/AuNPs solution was employed in the following experiments.

#### 3.4.3. Influence of the MbBA Concentration

The relationship between the peak current and the concentration of MbBA was also evaluated. When fixing the volume of MbBA to 30 μL, the peak current of ferrocene increased with the concentration of MbBA, which increased from 0.6 μM to 2.5 μM. The peak current of ferrocene then decreased with further increase of the concentration of MbBA (Figure 5D), indicating that less aptamer led to poor sensitivity and too much MbBA made it difficult for ferrocene to get close to the electrode surface, resulting in hindered electron transfer. From the plot of the current response versus the concentration of MbBA, the optimized amount of aptamer was determined as 2.5 μM.

### 3.5. Analytical Application of the Aptasensor

**Figure 6:**
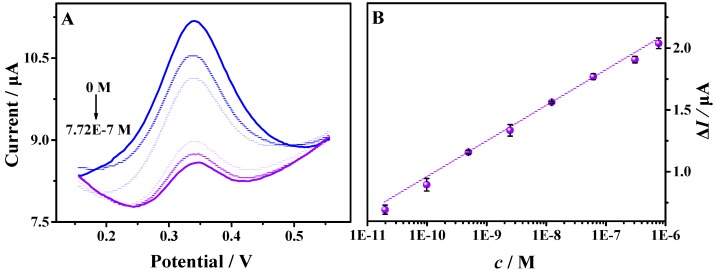
(**A**) DPVs of the biosensor for different concentrations of myoglobin: 0.00 M, 2.00 × 10^−11^ M, 1.00 × 10^−10^ M, 5.00 × 10^−10^ M, 2.50 × 10^−9^ M, 1.25 × 10^−8^ M, 6.18 × 10^−8^ M, 3.09 × 10^−7^ M, and 7.72 × 10^−7^ M in 5.0 mL 0.10 M PBS (pH = 7.4); (**B**) The calibration curve between the peak current changes and the myoglobin concentration (logarithm). The measurements were repeated 3 times to obtain the standard deviation. (Amplitude: 50 mV, pulse width: 0.050 s).

Under the optimum conditions, the electrochemical sensing performance of the MbBA/TCPP–Gr/AuNPs/GCE towards the myoglobin was studied by DPV. After incubating the sensing interface in different concentrations of myoglobin in PBS (pH = 7.4), the DPV signals of ferrocene were recorded. As seen in Figure 6, the calibration curve displayed a good linear relationship between the decreased peak current and the logarithm concentrations of myoglobin in the range from 2.0 × 10^−11^ M to 7.7 × 10^−7^ M (R^2^ = 0.9941). The linear regression equation is Δ*i* = 0.2882 lg*c* + 3.833. Here, Δ*i* is the decreased peak current and *c* is the concentration of myoglobin. The peak current decreased with the increase of myoglobin concentration. The reason was that the electron transfer rate was hindered after more targets were combined with MbBA. The limit of detection (LOD) was evaluated to be 6.7 × 10^−12^ M (S/N = 3), indicating a sensitive electrochemical aptasensor compared to most of the previous biosensors for myoglobin detection, as shown in Table 1. The excellent electrochemical-sensing performance may be attributed to large amounts of binding sites provided by AuNPs and the great electrocatalytic activity of graphene and AuNPs.

### 3.6. The Reproducibility, Stability and Specificity of the Aptasensor

The reproducibility of the aptasensor was examined by measuring 4.9 × 10^−10^ M myoglobin three times. The relative standard deviation (RSD) was 1.9%, indicating reasonable reproducibility. After the modified electrode was stored in refrigerator at 4 °C for 10 days, 98% of the initial peak current remained, which could be ascribed to the good stability of the electrode.

**Figure 7:**
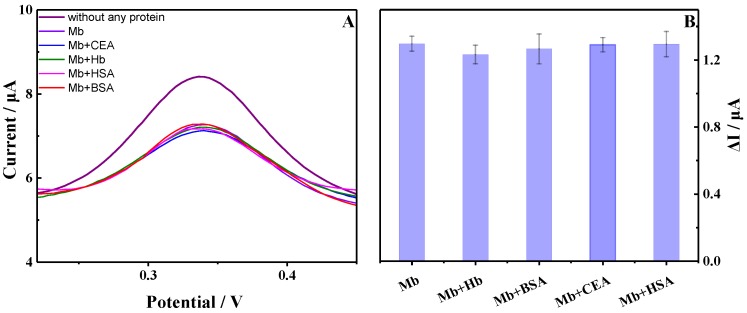
(**A**) DPVs of the aptasensor proposed in the absence and presence of unspecific proteins; (**B**) Specificity of the aptasensor for myoglobin detection. Error bars show the standard deviations of measurements taken from three times (Amplitude: 50 mV, pulse width: 0.050 s).

For evaluating the specificity of the developed aptasensor, some nontargets such as bovine serum albumin (BSA), human serum albumin (HSA), hemoglobin (Hb), and carcinoembryonic antigen (CEA) were tested. As shown in Figure 7, compared with the DPV signal of the proposed aptasensor immersed into 1.6 × 10^−9^ M myoglobin, the peak current changes are negligible after incubating the aptasensor in the mixture of myoglobin with the above four nontargets (50 times of the myoglobin concentration), respectively. It indicated the excellent specificity of the proposed aptasensor for myoglobin.

### 3.7. Real Sample Analysis

The validity and feasibility of the proposed electrochemical aptasensor was evaluated by recovery experiments using standard addition methods. Briefly, 50 μL of human serum was diluted to 5.0 mL with 0.10 M PBS (pH = 7.4), which is analogous to the patient serums. Then, different concentrations of myoglobin were added in diluted human serum samples. The detection steps were the same as those described above. As shown in Table 2, the recoveries are in the range of 96.8%–105.8%, illustrating good accuracy of the aptasensor for myoglobin analysis in real clinical samples.

## 4. Conclusions

In summary, we have successfully designed a sensitive and selective electrochemical aptasensor for myoglobin based on the newly synthesized TCPP–Gr/AuNPs. The nanocomposites were synthesized by a simple wet-chemical strategy and possessed the advantages of large surface area, good biocompatibility, excellent electric conductivity, and high stability. The attachment of TCPP on the graphene surface made the graphene disperse in aqueous solution uniformly. AuNPs provided a large number of binding sites for conjugating MbBA through a Au–S bond. The proposed aptasensor had a wide linear range and lower detection limit. It also showed good sensitivity, stability, and specificity. Besides, this method was cost-effective and easy to fabricate. In addition, the developed electrochemical aptasensor is promising for myoglobin detection in real clinical samples.

## Figures and Tables

**Table 1:** The comparison of different analytical methods for myoglobin detection.

Platform	Detection Method	Linear Range (nm)	Detection Limit (nm)	Specificity	Reference
3D RGO–Au/CILE	CV	200–36,000	60	No	[6]
Hemin/G-quadruplet/AuNPs	Colorimetric	0–1000	2.5	Antibody	[7]
Au/DSP/Peptide	CV	1.1–105	58	Myoglobin specific binding peptide	[12]
rGO/CNT	CV	0.058–235	0.020	Aptamer	[42]
POC	PGM	0–200	0.050	aptamer	[8]
CQDs	Fluorescence	0.059–5.9	0.059	Anti-Mb-aptamer	[43]
TCPP–Gr/AuNPs	DPV	0.020–770	0.0067	Aptamer	This work

**Table 2:** The recovery of myoglobin from human serum samples.

Samples	Added (nM)	Found (nM)	Recovery/%
1	0.152	0.159	104.6
2	1.56	1.51	96.8
3	9.88	10.5	106.3

## Abbreviations

The following abbreviations are used in this manuscript:

AMI	Acute Myocardial Infarction
AuNPs	Gold Nanoparticles
BSA	Bovine Serum Albumin
CEA	Carcinoembryonic Antigen
DPV	Differential Pulse Voltammetry
FT-IR	Fourier Infrared Spectrometer
GCE	Glassy Carbon Electrode
GO	Graphene Oxide
HAS	Human Serum Albumin
HAuCl_4_	Chloroauric Acid
HB	Hemoglobin
LOD	Limit of Detection
MbBA	Myoglobin Binding Aptamer
MCH	6-mercapto-l-hexanol
PBS	Phosphate Buffered Solution
PDDA	Poly Dimethyl Diallyl Ammonium Chloride
RSD	Relative Standard Deviation
SCE	Saturated Calomel Electrode
TCEP	Tris (2-carboxyethyl) Phosphine Hydrochloride
TCPP	Meso-tetra (4-carboxyphenyl) Porphyrin
TCPP-Gr	Meso-tetra (4-carboxyphenyl) Porphyrin Functionalized Graphene Composite
TEM	Transmission Electron Microscopy
UV-vis	Ultraviolet Visible Absorption Spectrum
XPS	X-ray Photoelectron Spectroscopy
